# Applying a RapidPlan model trained on a technique and orientation to another: a feasibility and dosimetric evaluation

**DOI:** 10.1186/s13014-016-0684-9

**Published:** 2016-08-18

**Authors:** Hao Wu, Fan Jiang, Haizhen Yue, Hui Zhang, Kun Wang, Yibao Zhang

**Affiliations:** 1 Key laboratory of Carcinogenesis and Translational Research (Ministry of Education/Beijing), Department of Radiotherapy, Peking University Cancer Hospital & Institute, Beijing, China; 2 Division of Ionizing Radiation Metrology, National Institute of Metrology, Beijing, 100029 China

**Keywords:** Knowledge-based planning, RapidPlan, Model application, VMAT, IMRT, Setup orientation

## Abstract

**Background:**

The development of a dose-volume-histogram (DVH) estimation model for knowledge-based planning is very time-consuming and it could be inefficient if it was only used for similar upcoming cases as supposed. It is clinically desirable to explore and validate other potential applications for a configured model. This study tests the hypothesis that a supine volumetric modulated arc therapy (VMAT) model can optimize intensity modulated radiotherapy (IMRT) plans of other patient setup orientations.

**Methods:**

Based on RapidPlan, a DVH estimation model was trained using 81 supine VMAT rectal plans and validated on 10 similar cases to ensure the robustness of its designed purpose. Attempts were then made to apply the model to re-optimize the dynamic MLC-sequences of the duplicated IMRT plans from 30 historical patients (20 prone and 10 supine) that were treated with the same prescription as for the model (50.6 and 41.8 Gy to 95 % of PGTV and PTV simultaneously/22 fractions). The performance of knowledge-based re-optimization and the impact of setup orientations were evaluated dosimetrically.

**Results:**

The VMAT model validation on similar cases showed comparable target dose distribution and significantly improved organ sparing (by 10.77 ~ 18.65 %) than the original plans. IMRT plans of either setup can be re-optimized using the supine VMAT model, which significantly reduced the dose to the bladder (by 25.88 % from 33.85 ± 2.96 to 25.09 ± 1.32 Gy for D_50 %_; by 22.77 % from 33.99 ± 2.77 to 26.25 ± 1.22 Gy for mean dose) and femoral head (by 12.27 % from 15.65 ± 3.33 to 13.73 ± 1.43 Gy for D_50 %_; by 10.09 % from 16.26 ± 2.74 to 14.62 ± 1.10 Gy for mean dose), all *P* < 0.01. Although the dose homogeneity and PGTV conformity index (CI__PGTV_) changed slightly (≤0.01), CI__PTV_ of IMRT plans was significantly increased (Δ = 0.17, *P* < 0.01) by the manually defined target-objectives in the VMAT optimizer. The semi-automated IMRT planning increased the global maximum dose and V_107 %_ due to the missing of hot spot suppression by specific manual optimizing or fluence map editing.

**Conclusions:**

The Varian RapidPlan model trained on a technique and orientation can be used for another. Knowledge-based planning improves organ sparing and quality consistency, yet the target-objectives defined for VMAT-optimizer should be readapted to IMRT planning, followed by manual hot spot processing.

## Background

Knowledge-based treatment planning is a promising solution to reduce the planning time [1–4] and planner-dependence of plan quality [5–11]. As a commercial knowledge-based optimizer, Varian RapidPlan (Varian Medical Systems, Palo Alto, CA) has been validated on patients with head & neck, lung, esophageal, breast, hepatocellular and prostate cancer [12–17] based on similar cases for model training and dosimetric testing. However, no attempts have been made to extend the domain of model applications to less-similar patients so far.

Considering the treatment techniques (VMAT/IMRT) and patient setup orientations (supine/prone) may vary even for the same disease, it would be very time-consuming if not impractical to train specific models for all clinical varieties. In addition, the existing similar plans of a special type may be insufficient for the configuration of a qualified model. Therefore, it is highly desirable to explore and validate other potential possibilities of using an existing model more efficiently. Considering the full-arc VMAT covers all possible field angles of IMRT, and the geometry-based expected dose (GED) algorithm of RapidPlan is independent from patient orientations, this study aims to investigate the feasibility and dosimetric performance of applying a DVH estimation model trained on supine VAMT plans to the knowledge-based optimization of IMRT plans of both supine and prone setup orientations.

## Methods

### Model configuration and validation

A DVH estimation model was configured with 81 simultaneous-integrated-boosting VMAT plans for pre-operative rectal cancer patients of supine setup. All training plans were manually created by senior dosimetrists based on Eclipse treatment planning system (V11.0 or before) following consistent dose prescriptions and planning protocols (50.6 Gy and 41.8 Gy to 95 % of PGTV and PTV respectively/ 22 fractions, 1–2 full arc, 5° collimator rotation, and 10 MV photon). As recommended by Varian’s manual [18], model validation was conducted on similar cases before publication. Specifically, 10 historical plans of the same type that were not used for the model training were duplicated, and the model was applied to re-optimize the plan copies (referred as RP-VMAT plans). The RP-VMAT plans were compared with the original plans to check if the model's robustness of its designed purpose was clinically acceptable.

### Knowledge-based IMRT planning

To avoid the bias of comparing the knowledge-based plans with the manual plans that were made suboptimal intentionally, the duplications of all 30 testing IMRT plans (20 prone and 10 supine, due to very limited supine patients treated with IMRT historically at our centre) were retrospectively selected from the clinically approved and treated cases of identical prescriptions as for the model. Using sliding window technique, each original IMRT plan was manually developed with five fields of 10 MV photon beams. The knowledge-based re-planning maintained all other settings except the MLC sequences were re-optimized using the estimates and objectives generated by the supine VMAT model (referred as RP-IMRT plans). The Photon Optimizer for IMRT (PO_13535), DVH Estimation Algorithm (v. 13.5.35) and Anisotropic Analytical Algorithm (AAA_13535) were selected for the automatic RP-IMRT optimization. Relative to the conventional DVO and PRO algorithms for the manual optimization of IMRT or VMAT plans, the new PO algorithm for RapidPlan is applicable to both techniques, which uses one single matrix over the image to define the structures, DVH calculation and dose sampling spatially [18]. Based on GED, PO partitions OAR voxels into four sub-volumes and predict the most likely landing range for the DVH curves, which were generated as optimization objectives for the knowledge-based planning. To base the dosimetric comparison on similar target dose coverage, all RP and original plans were renormalized to satisfy the dose prescriptions for both PGTV and PTV. Visual inspection of sectional dose distribution was routinely performed to examine the target coverage and hot spots appearance.

### Dosimetric assessment and statistical comparison

Using the following DVH metrics, the dosimetric features were compared between the original vs. RP-VMAT, original vs. RP-IMRT plans and prone vs. supine setup orientations respectively: 1. homogeneity index of PGTV (HI__PGTV_) and PTV (HI__PTV_), defined as (*D*_2 %_ − *D*_98 %_)/*D*_50 %_; 2. conformity index of PGTV (CI__PGTV_) and PTV (CI__PTV_), defined as *V*_100 %_/*V*_*t* arg *et*_; 3. the relative volume exceeding 107 % of the prescribed dose to PGTV (V_107 %_, i.e. V_54.14Gy_); 4. Global maximum dose (D_max_) and near maximum dose in PGTV (D_2 %_) [19]; 5. The dose to 50 % of the femoral head volume and urinary bladder volume (D_50 %_FH_ and D_50 %_UB_); 6. The mean dose to the femoral head and urinary bladder (D_mean_FH_ and D_mean_UB_). 7. The total monitor units (MUs) of each plan.

To assess the differences between the original plans and the knowledge based re-planning, paired samples *t*-test was conducted for normally distributed data (tested by Shapiro-Wilk method), otherwise Wilcoxon signed ranks test was performed using SPSS (version 21.0). To appraise the impact of supine and prone setup orientations on the dosimetric outcomes, independent sample *t*-test and Mann-Whitney *U* Test were carried out respectively for the data of normal and non-normal distributions. The equality of variances was examined by Levene's test. *P* < 0.05 was considered as statistically significant (2-tailed). Based on the tabular-formatted DVH data exported from Eclipse system, an in-house MATLAB code was programmed to calculate the mean DVHs of 30 patients that were either manually planned or knowledge-based re-planned. Plotting was performed using SigmaPlot software (Version 10.0, Systat, San Jose, CA).

## Results

### Model validation on similar cases

Table 1 displays the model validation results by optimizing similar supine VMAT cases. Relative to the manually optimized clinical plans, RapidPlan has brought negligible changes to HI__PGTV_, HI__PTV_, CI__PGTV_, CI__PTV_, D_max_ and D_2 %_. Negligible V_107 %_ values were observed in 3/10 original plans (magnitude ≤0.18 %), but only in 1/10 RP-VMAT population. On the other hand, RP-VMAT plans have significantly and largely relieved the normal organ exposure than the clinical plans.

**Table 1:** Validation of DVH estimation model by applying it to similar cases: dosimetric comparison between the 10 clinical supine VMAT plans and their knowledge-based re-optimizations

		Mean	SD	95 % Confidence intervals		△(%)	*P*
				Lower	Upper		
HI__PGTV_	Original	0.06	0.01	0.05	0.07	0.01	0.15*
	RP-VMAT	0.05	0.01	0.05	0.06		
HI__PTV_	Original	0.26	0.01	0.25	0.27	0	0.62*
	RP-VMAT	0.26	0.01	0.25	0.27		
CI__PGTV_	Original	1.01	0.04	0.98	1.04	0.01	0.11
	RP-VMAT	1.00	0.04	0.97	1.03		
CI__PTV_	Original	1.03	0.02	1.02	1.05	0.01	0.05*
	RP-VMAT	1.02	0.02	1.01	1.04		
D_max_	Original	53.93	0.35	53.69	54.18	0.36 (0.67 %)	0.07*
	RP-VMAT	53.57	0.50	53.22	53.93		
D_2 %_	Original	53.37	0.37	53.10	53.63	0.34 (0.64 %)	0.15*
	RP-VMAT	53.03	0.46	52.70	53.36		
D_50 %_UB_	Original	28.90	5.31	25.10	32.69	5.39 (18.65 %)	0.01
	RP-VMAT	23.51	3.26	21.18	25.84		
D_50 %_FH_	Original	16.30	1.66	15.11	17.48	2.30 (14.11 %)	0.01
	RP-VMAT	14.00	0.92	13.34	14.65		
D_mean_UB_	Original	30.01	4.52	26.77	33.24	4.51 (15.03 %)	0.01
	RP-VMAT	25.50	1.54	24.40	26.60		
D_mean_FH_	Original	17.18	1.80	15.90	18.47	1.85 (10.77 %)	0.01
	RP-VMAT	15.33	0.68	14.85	15.82		
MUs	Original	412	41	383	441	18 (4.37 %)	0.14
	RP-VMAT	430	23	413	447		

*Abbreviations*: *HI* homogeneity index, *CI* conformity index, *D*_*max*_ global maximum dose, *D*_*2 %*_ near maximum dose, *SD* standard deviation, *△(%)* difference between the original and RP-VMAT plans (% relative to the original value), *D*_*50 %*_ dose to the 50 % volume of the structure, *D*_*mea*n_ mean dose, *FH* femoral head, *UB* urinary bladder, *MU* monitor unit, *RP-VMAT* volumetric modulated arc therapy plans optimized by RapidPlan. Dose unit (Gy)

*Paired sample *T* test, otherwise Wilcoxon signed ranks test was used

### Original vs. RP-IMRT plans

**Fig. 1:**
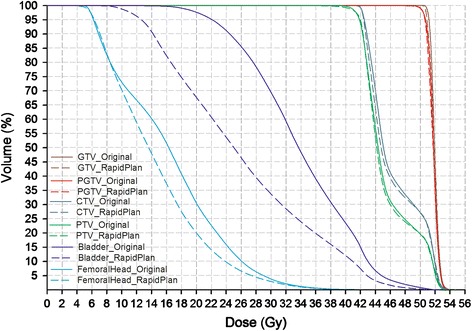
Mean DVHs of 30 IMRT patients planned by experience-based (original) or knowledge-based (RapidPlan) methods regardless of the setup orientations

Knowledge-based DVH estimations and objectives could be automatically generated for the RP-IMRT optimization using the VMAT model. As an explicit comparison between the original and RP-IMRT plans regardless of the setup orientations, Fig. 1 illustrates the average DVHs of the 30 patients stratified by the planning methods.

Relative to the original plans (Table 2), the model-assisted re-optimization has significantly reduced the dose to the urinary bladder and femoral head. The marginal changes of CI__PGTV_ and D_2 %_ were insignificant. Significant but negligible increases of HI were observed in RP-IMRT plans. However, knowledge-based IMRT planning using the VMAT model has significantly increased the dose conformity index to PTV and the global maximum dose. In addition, more cases (6/30 patients) with larger V_107 %_ values were observed in the RP-IMRT group (ranging from 0.01 ~ 1.76 %, not shown in Table 2) than in the original plans (hardly noticeable).

**Table 2:** Dosimetric comparison between the clinical IMRT plans (of both supine and prone setups) and their knowledge-based re-optimization using the DVH estimation model trained on supine VMAT plans

		Mean	SD	95 % Confidence intervals		△(%)	*P*
				Lower	Upper		
HI__PGTV_	Original	0.04	0.01	0.04	0.05	0.01	0.04
	RP-IMRT	0.05	0.01	0.04	0.05		
HI__PTV_	Original	0.25	0.01	0.24	0.25	0.01	0.01*
	RP-IMRT	0.26	0.01	0.25	0.26		
CI__PGTV_	Original	1.03	0.05	1.01	1.05	0.01	0.61
	RP-IMRT	1.02	0.05	1.00	1.04		
CI__PTV_	Original	1.01	0.02	1.00	1.02	0.17	<0.01*
	RP-IMRT	1.18	0.05	1.16	1.20		
D_max_	Original	52.95	0.44	52.78	53.11	1.51 (2.85 %)	<0.01
	RP-IMRT	54.46	1.52	53.89	55.02		
D_2 %_	Original	52.61	0.43	52.44	52.77	0.15 (0.29 %)	0.09
	RP-IMRT	52.76	0.51	52.57	52.95		
D_50 %_UB_	Original	33.85	2.96	32.74	34.95	8.76 (25.88 %)	<0.01*
	RP-IMRT	25.09	1.32	24.59	25.58		
D_50 %_FH_	Original	15.65	3.33	14.41	16.90	1.92 (12.27 %)	<0.01*
	RP-IMRT	13.73	1.43	13.19	14.26		
D_mean_UB_	Original	33.99	2.77	32.95	35.02	7.74 (22.77 %)	<0.01
	RP-IMRT	26.25	1.22	25.79	26.70		
D_mean_FH_	Original	16.26	2.74	15.24	17.28	1.64 (10.09)	<0.01
	RP-IMRT	14.62	1.10	14.21	15.04		
MUs	Original	805	82	774	836	394 (48.94 %)	<0.01*
	RP-IMRT	1199	80	1169	1229		

*Abbreviations*: *HI* homogeneity index, *CI* conformity index, *D*_*ma*x_ global maximum dose, *D*_*2 %*_ near maximum dose, *SD* standard deviation, *△(%)* difference between the original and RP-VMAT plans (% relative to the original value), *D*_*50 %*_ dose to the 50 % volume of the structure, *D*_*mean*_ mean dose, *FH* femoral head, *UB* urinary bladder, *MU* monitor unit, *RP-IMRT* intensity modulated radiotherapy plans optimized by RapidPlan. Dose unit (Gy)

*Paired sample *T* test, otherwise Wilcoxon signed ranks test was used

### Supine vs. prone setup orientations

Conflict was not encountered during the optimization of prone patients using the supine model. Table 3 compares the dosimetric outcomes between the supine and prone patients. Regarding the HI and CI of the targets, no or slight disparities were observed between the two setups for any planning methods. The absolute inter-orientation dose differences of D_max_, D_2 %_, D_50 %_UB_ and D_mean_UB_ were no more than 0.41 Gy. Prone patients received significantly lower D_50 %_FH_ and D_mean_FH_ in both original and RP-IMRT plans.

**Table 3:** Dosimetric statistics between the supine and prone IMRT patients

		Original					RapidPlan				
		Mean	SD	95 % CIs	△(%)	*P*	Mean	SD	95 % CIs	△(%)	*P*
HI__PGTV_	S.	0.04	0.01	0.04 ~ 0.05	0	0.66*	0.05	0.01	0.04 ~ 0.05	0	0.97
	P.	0.04	0.01	0.04 ~ 0.05			0.05	0.01	0.04 ~ 0.06		
HI__PTV_	S.	0.24	0.01	0.24 ~ 0.25	0.01	0.24*	0.25	0.01	0.25 ~ 0.26	0.01	0.22*
	P.	0.25	0.01	0.24 ~ 0.25			0.26	0.02	0.25 ~ 0.26		
CI__PGTV_	S.	1.03	0.07	0.99 ~ 1.09	0.01	0.73	1.01	0.06	0.97 ~ 1.05	0.01	0.48
	P.	1.02	0.05	1.00 ~ 1.04			1.02	0.05	0.99 ~ 1.04		
CI__PTV_	S.	1.02	0.02	1.00 ~ 1.04	0.01	0.16	1.19	0.07	1.14 ~ 1.24	0.01	0.56*
	P.	1.01	0.02	1.00 ~ 1.02			1.18	0.05	1.16 ~ 1.20		
D_max_	S.	52.93	0.45	52.61 ~ 53.25	0.03 (0.06 %)	0.98	54.73	1.67	53.53 ~ 55.93	0.41 (0.75 %)	0.48
	P.	52.96	0.45	52.75 ~ 53.17			54.32	1.46	53.64 ~ 55.00		
D_2 %_	S.	52.56	0.41	52.26 ~ 52.85	0.07 (0.13 %)	0.68*	52.71	0.24	52.53 ~ 52.88	0.08 (0.15 %)	0.86
	P.	52.63	0.45	52.42 ~ 52.84			52.79	0.61	52.50 ~ 53.07		
D_50 %_UB_	S.	34.13	2.73	32.16 ~ 36.06	0.41 (1.20 %)	0.74*	25.12	0.84	24.52 ~ 25.72	0.05 (0.20 %)	0.93*
	P.	33.72	3.12	32.26 ~ 35.18			25.07	1.53	24.36 ~ 25.79		
D_50 %_FH_	S.	18.89	2.69	16.96 ~ 20.81	4.85 (25.67 %)	<0.01*	14.67	1.80	13.38 ~ 15.95	1.41 (9.61 %)	0.01*
	P.	14.04	2.28	12.97 ~ 15.11			13.26	0.94	12.82 ~ 13.70		
D_mean_UB_	S.	34.25	2.90	32.17 ~ 36.32	0.39 (1.14 %)	0.72*	26.19	0.97	25.50 ~ 26.89	0.08 (0.31 %)	0.87*
	P.	33.86	2.77	32.56 ~ 35.15			26.27	1.36	25.64 ~ 26.91		
D_mean_FH_	S.	19.23	2.41	17.51 ~ 20.96	4.45 (23.14 %)	<0.01	15.41	1.07	14.64 ~ 16.17	1.18 (7.66 %)	<0.01*
	P.	14.78	1.31	14.17 ~ 15.39			14.23	0.91	13.81 ~ 14.66		

*Abbreviations*: *S.* supine, *P.* prone, *HI* homogeneity index, *CI* conformity index, *△(%)* difference between the supine and prone plans (% relative to the supine value), *D*_*ma*x_ global maximum dose, *D*_*2 %*_ near maximum dose, *SD* standard deviation, *95 % CI* 95 % confidence interval, *D*_*50 %*_ dose to the 50 % volume of the structure, *D*_*mean*_ mean dose, *FH* femoral head, *UB* urinary bladder. Dose unit (Gy)

*Independent sample *t*-test, otherwise Mann-Whitney *U* test was used

## Discussion

To appraise the contribution of the new PO optimizer, the RapidPlan-generated objectives were applied to the 10 VMAT validation plans using the old PRO optimizer, and the results were more close to the RP-VMAT than the original plans. Therefore, without changing any settings other than redesigning the MLC sequences, the dosimetric changes of knowledge-based re-optimization were mostly if not solely attributable to the estimates and objectives generated by the RapidPlan model based on patient-specific evaluations of structure sets and field geometry. However, as a key component of RapidPlan solution package, the minor role of the possible stronger PO algorithm cannot be excluded, which may deserve more investigations in the future.

Consistent with the published successful implementations of RapidPlan models to prospective patients of the same type [12–17],our supine VMAT model could generate clinically acceptable plans for similar validation patients as it was configured for. Comparable target dose homogeneity and conformity were achieved, but the dose to the critical organs was largely reduced than the clinical VMAT plans that were developed manually (Table 1). As a new attempt of extending the model application domain, significant improvement of critical organ sparing was also achievable by the model preconfigured on another technique and orientation (Table 2 and Fig. 1). Using the same model, RP-IMRT achieved greater improvement magnitudes of D_50 %_UB_ and D_mean_UB_ than RP-VMAT plans, yet the decreasing amplitudes of D_50 %_FH_ and D_mean_FH_ were slightly to the opposite. Therefore, the VMAT model did not necessarily work better in the similar technique than in the different, which can be very helpful in detecting and improving the sub-optimal manual IMRT plans. As a result of knowledge-based re-optimization of both IMRT and VMAT plans, the decreasing amplitudes of the dose to the urinary bladder were much larger than that to the femoral head. We ascribed this different magnitude to the greater geometric varieties of urinary bladder, which have made the experience-based subjective judgement of achievable goals more challenging in the conventional planning. Some unreliable decisions could be avoided by RapidPlan using the personalized quantitative evaluation algorithm, hence reducing unnecessary normal tissue complication risks associated with suboptimal planning [20]. Additional evidences of reducing inter-planner variety using knowledge-based planning were the smaller standard deviations and narrower 95 % confidence intervals of the dose metrics to the critical organs (Tables 1, 2 and 3), which is also consistent with pervious observations on other cancer types [5–11].

As shown in Fig. 1, the nearly overlapped lines of GTV, PGTV, CTV and PTV of the original and RP-IMRT plans indicate that the aforementioned comparisons of organ sparing are based on similar target dose coverage after renormalization. Slight but considerable sharper dose gradient in the transitional region from PGTV to PTV can be observed in the RP-IMRT plans (approximately between the dose range of 42 ~ 49 Gy), which can be ascribed to the fact that ‘PTV-(PGTV + 5 mm)’ was included as an ‘organ-at-risk’ (rather than a ‘target’) in the model configuration which generated upper constraints for this structure in the knowledge-based IMRT planning to shape a good dose fall-off. The volume of ‘PTV-(PGTV + 5 mm)’ was created by deducting PGTV and its 5 mm outer margin from PTV, which was also optimized during the manual planning.

As shown in Table 2, the significantly increased CI__PTV_ of RP-IMRT plans indicated deteriorated dose fall-off beyond the border of PTV, which might not be the ‘fault’ of the DVH-estimation model though. Indeed, the model itself does not generate knowledge-based predictions and estimations for the targets. Instead, these fixed objectives shall be manually assigned and can be incorporated into the optimizer as templates to facilitate an automated planning process. Although these combined parameters functioned well for the automated RP-VMAT planning, it was in line with our clinical experience that IMRT optimization usually adopts different constraints and priorities than the VMAT especially for the target dose coverage and hot volume control. Although this study focused on the feasibility and dosimetric evaluation of cross-applying the identical RP-VAMT optimizer, it is advisable to investigate the target-objective revision to better readapt the VMAT optimizer to the IMRT planning, and to test the capability of IMRT model in the knowledge-based VMAT planning in the future.

The increased D_max_ and V_107 %_ of RP-IMRT plans may be associated with the significantly more MUs than the original plans. As a comparison, the MU escalation of RP-VMAT plans was minor, where the hot spot did not increase considerably (it is also true that VMAT technique is less likely to produce hot spots than IMRT). Moreover, emerging hot spots were usually segmented timely and suppressed with high priority during the iteration of conventional optimization, and/or manually erased by editing the fluence map afterwards [21]. These steps were all missing during the semi-automated knowledge-based optimization. Therefore, manual examination and elimination of hot spot is highly recommended especially for other treatment regions involving serial organs at risk.

As shown in Table 3, the impact of setup orientations on the dosimetric outcomes of RP-IMRT plans was very tiny: the differences of HI__PGTV_, HI__PTV_, CI__PGTV_, CI__PTV_, D_max_, D_2 %_, D_50 %_UB_ and D_mean_UB_ between the prone and supine patients were negligible. The magnitudes of inter-setup variances were comparable to that of the original plans. The comparable results of knowledge-based IMRT planning between patients of opposite setups suggested that orientation variety did not affect the performance of a preconfigured model.

It was also noticed that the femoral head of prone patients received consistently lower dose for both original and RP-IMRT plans, but the difference may not be attributable to the orientation-disparity between the modelling and planning candidates, because the supine model has largely reduced the femoral exposure for both supine and prone patients. An alternative explanation was that some sub-optimal field geometry in the original supine IMRT plans may have involved more femoral head volume into the fields, hence induced more exposure for both original and RP-IMRT plans using identical field organizations. Nevertheless, RapidPlan reduced the magnitude of inter-setup dose disparities of D_50 %_FH_ and D_mean_FH_, suggesting that even under suboptimal field arrangement (which is not optimizable by RapidPlan), knowledge-based optimization still behaved superiorly in terms of plan quality and consistency.

## Conclusions

A supine VMAT model can automatically estimate optimization objectives for the knowledge-based IMRT planning of either supine or prone patients, yielding superior organ sparing and quality consistency than the conventional method. If incorporated as part of the optimizer, the manually added objectives and priorities for the targets should be adjusted in accordance with the selected treatment technique. Manual processing of the hot spots is highly recommended after the semi-automated knowledge-based IMRT planning.

## References

[1] Bolan C (2013). Expediting the treatment planning process. Appl Radiat Oncol..

[2] Zarepisheh M, Long T, Li N (2014). A DVH-guided IMRT optimization algorithm for automatic treatment planning and adaptive radiotherapy replanning. Med Phys.

[3] Moore K, Scott Brame R, Low D, Mutic S (2011). Experience based quality control of clinical intensity modulated radiotherapy planning. Int J Radiat Oncol Biol Phys.

[4] Appenzoller L, Michalski J, Thorstad W, Mutic S, Moore K (2012). Predicting dosevolume histograms for organs-at-risk in IMRT planning. Med Phys.

[5] Chanyavanich V, Das SK, Lee WR (2011). Knowledge-based IMRT treatment planning for prostate cancer. Med Phys.

[6] Zhu X, Ge Y, Li T (2011). A planning quality evaluation tool for prostate adaptive IMRT based on machine learning. Med Phys.

[7] Wu B, McNutt T, Zahurak M (2012). Fully automated simultaneous integrated boosted-intensity modulated radiation therapy treatment planning is feasible for head-and-neck cancer: a prospective clinical study. Int J Radiat Oncol Biol Phys.

[8] Lian J, Yuan L, Ge Y (2013). Modeling the dosimetry of organ-at-risk in head and neck IMRT planning: an inter technique and inter institutional study. Med Phys.

[9] Wu B, Pang D, Simari P (2013). Using overlap volume histogram and IMRT plan data to guide and automate VMAT planning: a head-and neck case study. Med Phys.

[10] Yang Y, Ford EC, Wu B (2013). An overlap-volume-histogram based method for rectal dose prediction and automated treatment planning in the external beam prostate radiotherapy following hydrogel injection. Med Phys.

[11] Good D, Lo J, Lee WR (2013). A knowledge-based approach to improving and homogenizing intensity modulated radiation therapy planning quality among treatment centers: an example application to prostate cancer planning. Int J Radiat Oncol Biol Phys.

[12] Fogliata A, Belosi F, Clivio A (2014). On the pre-clinical validation of a commercial model-based optimisation engine: Application to volumetric modulated arc therapy for patients with lung or prostate cancer. Radiothera Oncol.

[13] Nwankwo O, Mekdash H, Sihono DSK (2015). Knowledge-based radiation therapy (KBRT) treatment planning versus planning by experts: validation of a KBRT algorithm for prostate cancer treatment planning. Radiat Oncol.

[14] Fogliata A, Nicolini G, Clivio A (2015). A broad scope knowledge based model for optimization of VMAT in esophageal cancer: validation and assessment of plan quality among different treatment centers[J]. Radiat Oncol.

[15] Fogliata A, Nicolini G, Bourgier C (2015). Performance of a knowledge-based model for optimization of volumetric modulated arc therapy plans for single and bilateral breast irradiation[J]. PLoS One.

[16] Tol JP, Delaney AR, Dahele M (2015). Evaluation of a knowledge-based planning solution for head and neck cancer. Int J Radiat Oncol Biol Phys.

[17] Fogliata A, Wang PM, Belosi F (2014). Assessment of a model based optimization engine for volumetric modulated arc therapy for patients with advanced hepatocellular cancer. Radiat Oncol.

[18] Varian. Treatment planning 13.5 new features | RapidPlan. EC13.5-WBK-01-B. Palo Alto; 2014.

[19] ICRU (2010). Prescribing, recording and reporting photon-beam intensity-modulated radiation therapy (IMRT). J ICRU.

[20] Moore KL, Schmidt R, Moiseenko V (2015). Quantifying unnecessary normal tissue complication risks due to suboptimal planning: A secondary study of RTOG 0126. Int J Radiat Oncol Biol Phys.

[21] Cook JT, Tobler M, Leavitt DD (2006). IMRT fluence map editing to control hot and cold spots. Med Dosim.

